# Augmented sphingosine 1 phosphate receptor-1 signaling in cardiac fibroblasts induces cardiac hypertrophy and fibrosis through angiotensin II and interleukin-6

**DOI:** 10.1371/journal.pone.0182329

**Published:** 2017-08-03

**Authors:** Sei-ichiro Ohkura, Soichiro Usui, Shin-ichiro Takashima, Noriko Takuwa, Kazuaki Yoshioka, Yasuo Okamoto, Yutaka Inagaki, Naotoshi Sugimoto, Teppei Kitano, Masayuki Takamura, Takashi Wada, Shuichi Kaneko, Yoh Takuwa

**Affiliations:** 1 Department of Physiology, Kanazawa University School of Medicine, Ishikawa, Japan; 2 Department of System Biology, Kanazawa University School of Medicine, Ishikawa, Japan; 3 Department of Health and Medical Sciences, Ishikawa Prefectural Nursing University, Ishikawa, Japan; 4 Center for Matrix Biology and Medicine, Graduate School of Medicine, Tokai University, Kanagawa, Japan; 5 Department of Nephrology and Laboratory Medicine, Kanazawa University School of Medicine, Ishikawa, Japan; Rutgers New Jersey Medical School, UNITED STATES

## Abstract

Background: Cardiac fibroblasts, together with cardiomyocytes, occupy the majority of cells in the myocardium and are involved in myocardial remodeling. The lysophospholipid mediator sphigosine-1-phosphate (S1P) regulates functions of cardiovascular cells through multiple receptors including S1PR1–S1PR3. S1PR1 but not other S1P receptors was upregulated in angiotensin II-induced hypertrophic hearts. Therefore, we investigated a role of S1PR1 in fibroblasts for cardiac remodeling by employing transgenic mice that overexpressed S1PR1 under the control of α-smooth muscle actin promoter. In S1PR1-transgenic mouse heart, fibroblasts and/or myofibroblasts were hyperplastic, and those cells as well as vascular smooth muscle cells overexpressed S1PR1. Transgenic mice developed bi-ventricular hypertrophy by 12-week-old and diffuse interstitial fibrosis by 24-week-old without hemodynamic stress. Cardiac remodeling in transgenic mice was associated with greater ERK phosphorylation, upregulation of fetal genes, and systolic dysfunction. Transgenic mouse heart showed increased mRNA expression of angiotensin-converting enzyme and interleukin-6 (IL-6). Isolated fibroblasts from transgenic mice exhibited enhanced generation of angiotensin II, which in turn stimulated IL-6 release. Either an AT1 blocker or angiotensin-converting enzyme inhibitor prevented development of cardiac hypertrophy and fibrosis, systolic dysfunction and increased IL-6 expression in transgenic mice. Finally, administration of anti-IL-6 antibody abolished an increase in tyrosine phosphorylation of STAT3, a major signaling molecule downstream of IL-6, in the transgenic mouse heart and prevented development of cardiac hypertrophy in transgenic mice. These results demonstrate a promoting role of S1PR1 in cardiac fibroblasts for cardiac remodeling, in which angiotensin II—AT1 and IL-6 are involved.

## Introduction

Increasing evidence indicates that cardiac hypertrophy is an independent risk factor for the development of heart failure [1]. Initially, cardiac hypertrophy is a physiological adaptation of the heart against increased workload to maintain normal heart function. However, sustained pathological hypertrophic stimuli induce cardiomyocyte apoptosis and interstitial fibrosis, which result in cardiac dysfunction [2]. Besides mechanical stresses, several neurohumoral factors acting via G protein-coupled receptors, which include angiotensin II (Ang II), endothelins and catecholamines, have been identified as potent inducers of cardiac hypertrophy [3].

Sphingosine-1-phosphate (S1P), a lysophospholipid mediator, exerts pleiotropic actions including cell proliferation, migration, differentiation and cell-cell adhesion [4]. The actions of S1P are mediated by S1P-specific G protein-coupled receptor family comprising S1PR1 to S1PR5. Cardiomyocytes express S1PR1, S1PR2, and S1PR3. An early study [5] in neonatal rat cardiomyocytes demonstrated that S1P did not induce hypertrophy in vitro although structurally related sphingosylphosphorylcoline was able to induce hypertrophy, whereas other studies [6] showed that S1P induced hypertrophy of rat and mouse cardiomyocytes via S1PR1. However, there have been only a few in vivo studies that explored roles of S1P receptors in cardiac remodeling [7]. We recently reported that mice that overexpress the S1P-synthesizing enzyme sphingosine kinase (SphK)-1 developed progressive myocardial fibrosis without hypertrophy, suggesting a role of S1P for cardiac remodeling in vivo [8].

Non-myocytes, predominantly fibroblasts, account for 60–70% of the cell number in the myocardium although cardiomyocytes occupy the bulk of myocardial mass, and are critical to maintain normal heart function through controlling extracellular matrix turnover and serving electro-mechanical functions [9]. Cardiac fibroblasts release paracrine and autocrine mediators, which include endothelin-1, Ang-II, tumor necrosis factor-α and transforming growth factor-β (TGF-β), into the microenvironment. These factors act on cardiomyocytes, inflammatory cells and fibroblasts themselves, contributing to cardiac remodeling. The phenotype of fibroblasts can change from a quiescent one in the normal heart to a proliferative, migratory and secretory one as observed, for example, after myocardial infarction [10]. Fibroblasts, which exhibit the latter phenotype and express contractile proteins including α-smooth muscle actin (αSMA), are termed myofibroblasts and implicated in fibrosis in various organs including heart.

Cardiac fibroblasts express S1PR1, S1PR2 and S1PR3 [11]. Among these, S1PR1 is coupled exclusively to Gi to stimulate Ras/extracellular signal-regulated kinase (ERK), phsphoinositide 3-kinase/Akt and Rac pathways, which promote cell proliferation and migration. We observed that S1PR1 was expressed in cardiac fibroblasts and upregulated in the hypertrophic heart of mice receiving Ang II infusion. Previous study shows that Ang II increases the expression of αSMA in fibroblast [12]. Ang II infusion also increased αSMA expression in hearts. Therefore, we generated mice that overexpress S1PR1 transgene under the control of αSMA promoter to study the role of S1PR1 in cardiac fibroblast during cardiac remodeling in vivo. We found in the transgenic (TG) mice that augmented S1PR1 signaling in myofibroblasts led to the development of pathological cardiac hypertrophy with interstitial fibrosis, which was mediated through the paracrine actions of Ang II and interleukin-6 (IL-6) released by myofibroblasts. The transgenic mice provide a new model of Ang II-dependent hypertrophy without pressure overload.

## Materials and methods

### Generation of transgenic mice overexpressing S1PR1 (S1PR1-TG mice)

The expression plasmid that drives S1PR1 expression under αSMA promoter was constructed as follows: Approximately 4700-bp EcoRI-BamHI fragment of human αSMA promoter DNA from pBS-HSMA-EA4.7 (kindly donated by Dr. T. Miwa in Osaka University) was ligated onto the mammalian expression vector pCAGGS, in which the unique Hind III site had been replaced by Not I site, at EcoRI site. The 1500-bp Stu I-Bgl II fragment of rat S1PR1 cDNA encoding the entire coding sequence, which was blunted and Bst XI adaptor-ligated, was ligated onto pCAGGS at Bst XI site. The ~8500bp linearized Sal I-Not I fragment of the resultant expression construct was injected into fertilized oocytes from superovulated C57BL6/J mice according to the standard procedure. The injected oocytes were transferred to the oviducts of pseudopregnant mice. The F0 generation was screened for the integration of the transgene by polymerase chain reaction (PCR) of tail genomic DNA with the sense primer 5’-AAC CGG AAG CTG TTG ATA CTG and the antisense primer 5’-CTC AGT GGT ATT TGT GAG CCA, which amplified 315-bp DNA product. TG mice were mated with C57BL6/J mice and three lines were established. Mice were housed in a temperature-controlled conventional facility (24°C) under a 12:12h light-dark cycle with free access to regular chow and water at Institute for Experimental Animal, Advanced Science Research Center, Kanazawa University. Col1α2-EGFP mice were generated as previously described [13]. To obtain S1PR1-overexpressing ColIα2-EGFP transgenic mice, S1PR1-TG mice and ColIα2-EGFP TG mice were crossed. All procedures were conducted in accordance with Fundamental Guidelines for Proper Conduct of Animal Experiment and Related Activities in Academic Research Institutions under the jurisdiction of the Ministry of Education, Culture, Sports, Science and Technology of Japan approved by Committee on Animal Experimentation of Kanazawa University (Approval NO. 44103–1).

### Chronic treatment of mice with pharmacological agents

Six week-old TG mice were given candesartan (CDS) (10mg/kg/day in drinking water, a gift from Takeda Pharmaceutical Company (Osaka, Japan)) or cilazapril (10mg/kg/day in drinking water, a gift from Eisai. Co. Ltd. (Tokyo, Japan)) for 18 weeks. At the end of the administration period, mice were subjected to echocardiography and then euthanized by an excess dose of pentobarbital, followed by morphological analysis of cardiac hypertrophy and fibrosis. In chronic Ang II infusion experiments, a subpressor dose of Ang II (0.3 mg/kg/day) was administered into 12-week-old C57BL6/J wild-type mice for 14 days by using osmotic mini-pump (Alzet, Cupertino, CA). Physiological saline was administered into control mice. For in vivo IL-6 neutralization, mice received intraperitoneal injections of 100μg rat anti-mouse IL-6 mAb (clone MP5-20F3) (R&D Systems) in 0.2 ml Dulbecco’s phosphate-buffered saline (PBS). Rat IgG1 (clone R3-34) (R&D Systems) was administered as isotype-matched control. Administration of antibodies was started at 8-weeks and conducted every two weeks until 12-weeks.

### Blood pressure measurement and echocardiography

Systolic and diastolic blood pressure of conscious mice was non-invasively measured by a tail cuff method using Softron BP98A (Softron Co. Ltd., Tokyo, Japan) as described previously. Mice that were anesthetized with pentobarbital (60 mg/kg) underwent echocardiography using an ultrasonography (Agilent Technology SONOS 5500). A 12-MHz linear ultrasound transducer (S 12) was applied to depilated left anterior chest wall. M-mode measurements of the left ventricular (LV) internal diameter, the interventricular septal dimension (IVSd), and the posterior wall dimension (PWd) were taken from more than five beats and averaged. LV end-diastolic diameter (EDD) was measured at the time of the apparent maximal LV diastolic dimension. LV end-systolic diameter (ESD) was measured at the time of the most anterior systolic excursion of the posterior wall. Percent fractional shortening (%FS) was calculated as (EDD-ESD)/EDD x 100.

### Northern blot analysis and quantitative real-time polymerase chain reaction (PCR)

Total RNA was isolated from various organs by using RNeasy (Qiagen). The mRNA expression of S1PR1, S1PR2, S1PR3, atrial natriuretic peptide (ANP), brain natriuretic peptide (BNP), β-myosin heavy chain (βMHC), collagen I α-1 chain, skeletal muscle α-action (αSKMA), and glyceraldehyde 3-phosphate dehydrogenase (GAPDH) as an internal control was analyzed by Northern analysis as described previously. The expression of S1PR1, S1PR2 and S1PR3, was also analyzed by conventional reverse transcription-PCR as described previously. Real-time Quantitative PCR analysis was performed using the ABI PRISM 7300 sequence detection system (Applied Biosystems, Foster, CA). The following primers and TaqMan probes (Applied Biosystems) were used: sphingosine kineses (SphK)-1 (Sphk1, Mm00448841_g1) and -2 (Sphk2, Mm00445021_m1), S1PR1 (S1pr1, Mm02619656_s1), S1PR2 (Edg5, Mm02620208_s1), S1PR3 (S1pr3, Mm02620181_s1), angiotensinogen (Agt, Mm00599662_m1), angiotensin converting enzyme (Ace, ID# Mm00802048_m1), AT_1_ (Agtr1, ID# Mm00507771_m1), AT_2_ (Agtr2, ID# Mm01341373_m1), IL-6 (Il6, Mm99999064_m1), endothelin-1 (Edn1, Mm00438656_m1), insulin-like growth factor-I (IGF-I) (Igf-1, Mm00439560_m1), cardiotrophin-1 (CT-1, Mm00432772_m1), leukemia inhibitory factor (LIF) (Lif, Mm00434762_g1), LIF receptor (LIFR) (Lifr, Mm00442942_m1), GP130 (Lrpprc, Mm00511512_m1), and transforming growth factor-β1 (TGF−β1) (Tgf-β1, Mm00441726_m1). Glyceraldehyde 3-phosphate dehydrogenase (GAPDH) (Gapdh, Mm03302249_g1) was used as an endogenous control. The cycling condition was programmed as follows: activation of AmpErase uracil-N-glycosylase for prevention of carryover contamination at 50°C for 2 min, activation of AmpliTaq Gold DNA polymerase at 95°C for 10 min, 40 cycles of denaturation at 95°C for 15 s and annealing/extension at 60°C for 1 min. ΔCt was calculated as (gene of interest Ct)−(GAPDH Ct) using Sequence detector (Applied Biosystems). The relative quantity of mRNA of gene of interest was calculated by ΔΔCt calculation as 2^−((ΔCt of treated sample)− (ΔCt of control sample))^. The amplification efficiencies of the target and the endogenous reference were confirmed by observing the equal relationship between cDNA dilution and ΔCt. All experiments included negative controls consisting of no cDNA for each primer pair.

### Western blot analysis

The homogenates of hearts were prepared in ice cold heart homogenizing buffer containing 150 nmol/L NaCl, 5 mmol/L EDTA, 50mmol/L Tris/HCl (pH 7.5), 1 mmol/L Na3VO4, 10mmol/L NaF, 1% Triton X 100, 10% glycerol, 1mmol/L phenylmethylsulfonylfluoride and 10mg/ml each of leupeptin and aprotinin. After centrifugation at 3000 x g, the supernatant devoid of nuclei and debris were centrifuged at 10,000 x g for 10 min. The resultant supernatant was mixed with 4 x laemmli’s sample buffer at the ratio of 3:1, followed by the separation on the 10% SDS-polyacrylamide gel electrophoresis. Separated proteins were electro-transferred onto Immobilon-P (Millipore), and probed by rabbit polyclonal anti-Thr^202^, Tyr^204^-phosphorylated-ERK antibody (1:1000, Cell Signaling), rabbit polyclonal anti-ERK antibody (1:1000, Cell Signaling), rabbit polyclonal Tyr^705^-phosphorylated-STAT3 (1:1000 Cell Signaling) and rabbit S1PR1 C-terminus (1:1000, a kind gift from Dr. S. Mandala at Merck). The band density of ERKs (p42 and p44) was quantified by using Image J software (NIH), corrected for that of total ERK, and expressed as multiples of the values in control groups, which was expressed as 1.0.

### Isolation of mouse cardiac fibroblasts and myocytes

Mouse cardiac fibroblasts and myocytes were isolated by enzymatic digestion with collagenase and trypsin. Briefly, 2 hearts were isolated and dissected free of vessels and atria. The cannula was inserted into ascending aorta, and the hearts were perfused for 4 minutes with the collagenase buffer that contained (in wt/vol) 0.3% collagenase type II (Worthington), and 0.05% trypsin (Worthington) in Krebs buffer at 37°C. The digested heart tissues were finely fragmented by gentle pipetting and filtered by using a mesh (BD Biosciences). The cell suspensions were centrifugated at 400 x g and resuspended in Dulbecco’s modified Eagle’s minimal essential medium (DMEM) containing 10% fetal bovine serum. The suspended cells were plated, and 1 h later non-adherent cells were collected. Adherent cardiac fibroblasts were expanded by dispersing with trypsin/EDTA solution on reaching confluence and replating at the split ratio of 1:4. Characteristic fibroblasts morphology was visually confirmed under a light microscope. Nearly 100% of the cells were positive for anti-vimentin staining. Because the phenotype of fibroblasts can be influenced by the growth condition including the passage number, fibroblasts at the passage 1 to 3 were used for the present experiments.

### Histological analysis, immunohistochemistry, and immunofluorescence

Mice were euthanized by intraperitoneally injecting an excess dose of pentobarbital. Serial horizontal sections of acetone-fixed, fresh-frozen sections of hearts were subjected to immunofluorescence using anti-αSMA (clone 1A4, Sigma), anti-S1PR1, anti-CD31, and anti-GFP antibodies. The samples were examined using an inverted fluorescence microscope (Olympus IX70) under a PlanApo ×40/NA0.95 objective or a confocal laser-scanning microscope (Zeiss Axiovert 200M with LSM5 Pascal) equipped with a PlanApo ×63/NA1.4 oil-immersion objective. The mean cross-sectional cardiomyocyte areas were calculated by measuring 100 cells per specimen that had been subjected to silver impregnation staining. Fibrotic areas were determined by calculating Azan staining—positive area over total areas by using Image J software.

### Determination of Ang II and IL-6 in the conditioned media of cardiac fibroblasts

The serum-free culture media (2ml per dish) were collected from dishes with or without treatment of the cells with S1P (100nmol/L) and CDS (1μmol/L) for 24 h. Ang II concentration was determined by radioimmunoassay using two antibodies specific for Ang II (SRL Co., Hachioji, Japan). IL-6 concentrations were determined with an ELISA kit (R&D Systems).

### Statistics

**Fig 1:**
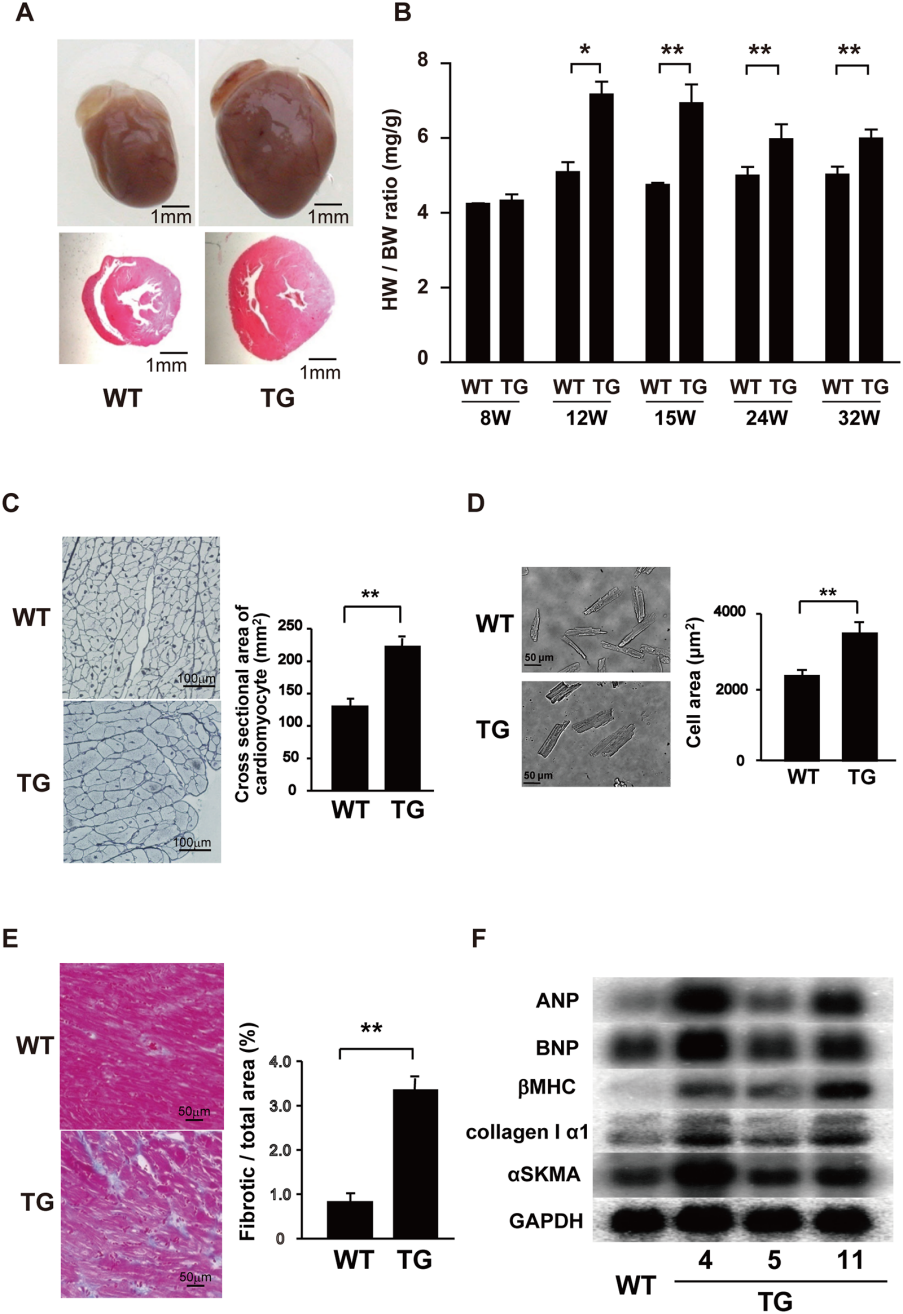
Transgenic overexpression of S1PR1 induces pathological cardiac hypertrophy. (A) Gross views and HE-stained cross-sections of the hearts from WT and TG mice. (B) The HW/BW ratio in 8- to 32-week-old WT and TG mice. n = 7 mice per group. *, p<0.05 and **, p<0.01. (C) Silver-staining of myocardial sections of 12-week-old WT and TG mice (right). Quantified data of mean cross sectional area of cardiomyocytes (left). (D) Images of representative isolated cardiomyocytes from WT and TG mice at 12 weeks (left). Quantification of cell areas of isolated cardiomyocytes (right). (E) Azan staining of myocardial sections of 24-week-old WT and TG mice (left). Quantified data of fibrotic areas in Azan stained sections of the hearts (right). (F) Northern blot analysis of mRNAs of natriuretic peptides ANP and BNP, fetal contractile proteins βMHC and αSKA and type I collagen α1 chain.

All data are shown as means +/- SEM. One-way ANOVA was followed by a post hoc Bonferroni-Dunn’s comparison test to determine statistical significance of differences between mean values. Unpaired t-test was performed for the comparison between two groups in Fig 1B, 1C, 1D and 1E, S1 Table, and S1, S4 and S6B Figs. For all statistical comparisons, p<0.05 was considered significant.

## Results

### αSMA promoter-driven S1PR1 overexpression induced cardiac hypertrophy and fibrosis with impaired cardiac function

Chronic Ang II infusion into wild-type (WT) mice increased the cardiac mRNA expression of S1PR1, SphK1 and αSMA but not S1PR2, S1PR3 or SphK2 (S1 Fig). The observations prompted us to investigate a role of S1PR1 in cardiac remodeling. We generated TG mice overexpressing S1PR1 under the control of the αSMA promoter and established three independent TG mouse lines (line 4, 5 and 11). Analysis of S1PR1 mRNA expression in various organs of line 4 TG mice shows that S1PR1 transgene mRNA is expressed in most of organs including lung, intestine, heart and kidney (Panel A in S2 Fig). The expression levels of S1PR1 transgene in hearts of line 4 and 11 mice were slightly higher compared with endogenous S1PR1, but that in line 5 mice was much lower ((Panel B in S2 Fig). Accordingly, the protein level of S1PR1 in heart of line 4 was greater than in WT mice (Panel C in S2 Fig). Anti-S1PR1 immunohistochemical staining showed enhanced S1PR1 expression in the vascular wall of heart and interstitial cells compared with WT mice (Panels D-G in S2 Fig), S1PR1 expression was increased in the aortic media, and the smooth muscle layers of bronchi, intestine, urinary bladder and uterus of TG mice compared with WT mice (Panel H in S2 Fig). Line 4 mice were employed for most of the analyses in the following study.

All three lines of TG mice developed postnatal bi-ventricular cardiac hypertrophy, which became maximal by 15-weeks (Fig 1A and 1B), with hypertrophy being more severe in lines 4 and 11 than in line 5 TG mice. Cardiac hypertrophy in TG mice was diffuse with hypertrophy of the interventricular septum and the free wall of both right and left ventricles. Individual cardiomyocytes in TG mice were hypertrophic compared with those from WT mice (Fig 1C). Cardiomyocytes isolated from TG mice displayed greater cell area compared with those from WT mice (Fig 1D). In TG heart at 24-weeks but not 12 weeks, increased diffuse accumulation of collagen fibers in both interstitial and perivascular areas was observed compared with WT mice (Fig 1E). Consistent with these pathological changes in TG heart, the mRNA expression of collagen I α-1 chain and the embryonic genes, which include atrial natriuretic peptide (ANP), brain natriuretic peptide (BNP), β-myosin heavy chain (β-MHC) and skeletal muscle α-actin, was elevated markedly in lines 4 and 11 TG mice and modestly in line 5 compared with WT mice (Fig 1F). Echocardiographic determinations showed a decreased %fractional shortening (%FS) without ventricular dilatation in line 4 mice at 24-weeks but not 12-weeks compared with line 4 TG mice (S3 Fig), indicating impaired contractile function in TG mice. TG mice were normotensive at 12-weeks, suggesting that ventricular hypertrophy was not a result of pressure overload (S1 Table). The mean life-span of TG mice was not different from that of WT mice up to 24-weeks.

### Cardiac fibroblasts are non-vascular cells that overexpress S1PR1 in heart of TG mice

**Fig 2:**
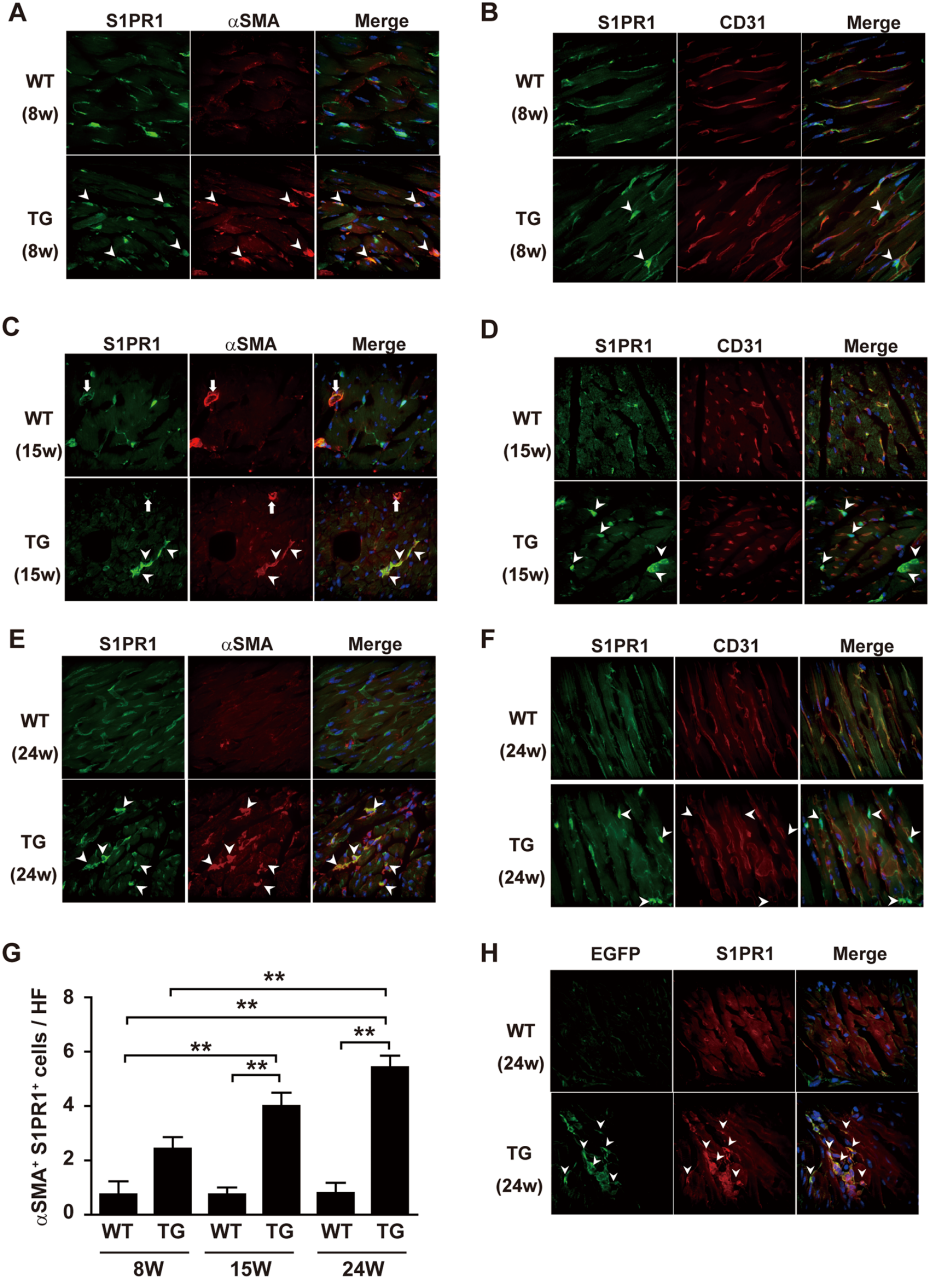
S1PR1 is expressed in fibroblasts in heart of TG mice. Double immunofluorescence staining of ventricular sections from 8- (A, B), 15- (C, D), and 24-week-old (E, F) WT and TG mice. (A, C and E) Anti-αSMA and anti-S1PR1 staining. In both WT and TG mice, S1PR1 is diffusely expressed in αSMA-negative cells. However, some scattered and solitary S1PR1- and αSMA-double positive apparently non-vascular wall cells (arrowheads) are observed in TG mice but not WT mice, and these double positive non-vascular wall cells increase with aging in TG mice. The arrows in C denote S1PR1- and αSMA-double positive vascular wall cells in WT and TG mice. (B, D and F) Anti-S1PR1 and anti-CD31 staining. In both WT and TG mice, S1PR1 is diffusely expressed in CD31-positive vascular endothelial cells. However, some S1PR1-positive cells are CD31-negative (arrowheads) and these S1PR1-positive and CD31-negative cells increase with aging in TG mice. (G) Quantified data of scattered and solitary αSMA- and S1PR1-positive cells. HF, high power field. **, p<0.01. (H) Anti-EGFP and anti-S1PR1 double immunofluorescence staining of ventricular sections from 24-week-old WT and S1PR1-TG mice with the ColI α2-EGFP background. S1PR1 is overexpressed in EGFP-positive cells (arrowheads) in TG mice but not WT mice.

We determined S1PR1 expression in the heart in both WT and TG mice at three stages, namely the pre-hypertrophic stage (8-week-old), the hypertrophic stage (15-week-old), and the hypertrophic and fibrotic stage (24-week-old), using anti-S1PR1 immunofluorescent staining. At 8-weeks, we detected many S1PR1-positive cells diffusely in the myocardium of both WT and TG mice (Fig 2A). Because S1PR1 is known to be expressed in vascular endothelial cells, we performed anti-S1PR1 and anti-CD31 (endothelial marker) double immunofluorescent staining. Virtually all S1PR1-positive cells in WT mouse heart are CD31-positive endothelial cells (Fig 2B). In TG mouse heart, most S1PR1-positive cells are CD31-positive. However, some S1PR1-positive cells are CD31-negative (arrowheads in Fig 2B) and show different morphology from CD31- and S1PR1-double positive endothelial cells. Anti-S1PR1 and anti-αSMA double immunostaining showed that S1PR1- and αSMA-double positive cells are observed in TG heart (arrowheads in Fig 2A). These double positive cells are scattered and solitary, and so do not look like vascular wall cells. Therefore, these αSMA-positive cells are most likely myofibroblasts. Thus, at the earlier prehypertrophic stage, some fibroblasts are already activated as suggested by their αSMA expression and exhibit the αSMA promoter-driven overexpression of S1PR1. At 15-weeks, the S1PR1-positive but CD31-negative cells are more frequently observed in TG heart compared with 8-week-old TG heart, but hardly seen in WT heart (Fig 2D). The scattered and solitary S1PR1- and αSMA-double positive cells are observed in 15-week-old TG heart (Fig 2C). Less frequently, S1PR1- and αSMA-double positive cells were observed in the vascular wall of arterioles in both WT and TG hearts (Fig 2C). These double positive cells in the vascular wall are likely smooth muscle or pericytes. At 24-weeks, the S1PR1-positive but CD31-negative cells, and the scattered type of S1PR1- and αSMA -double positive cells in TG heart are more abundant compared with 8- and 15-weeks (Fig 2E, 2F and 2G), and they are often clustered (Fig 2E, arrowheads). In 24-week-old S1PR1-TG mice with the ColI α2-EGFP background, EGFP-positive fibroblasts were intensely S1PR1-positive (Fig 2H), providing further evidence that cardiac fibroblasts overexpress S1PR1 in TG mice.

### Cardiac hypertrophy in TG mice is dependent on Ang II-AT1

**Fig 3:**
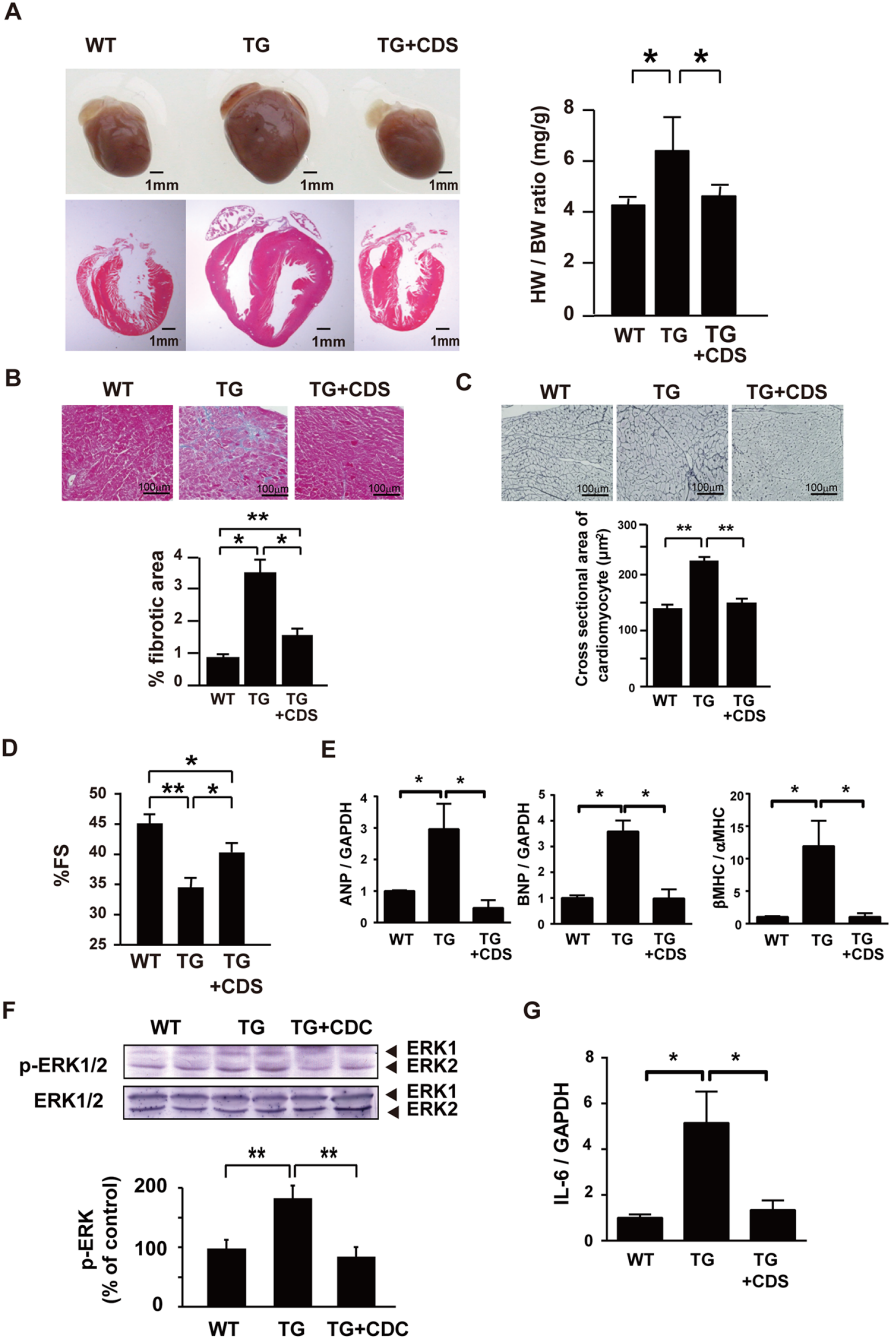
An AT1 blocker inhibits ventricular hypertrophy and fibrosis in TG mice. Administration of the AT1 blocker candesartan (CDS) was started at 6-weeks and continued for 18 weeks. (A) Gross views and HE-stained sections of the hearts from WT, non-treated TG, and CDS-treated TG mice at 24-week-old (left panel), and the HW/BW ratio in each mouse group (right panel). n = 6 mice per group. (B) Azan staining of myocardial sections of WT, non-treated TG, and CDS-treated TG mice at 24-weeks (upper panel) and quantified data (lower panel). n = 6 per group. (C) Silver-staining of myocardial sections of WT, non-treated TG, and CDS-treated TG mice at 24-weeks (upper panel) and quantified data (lower panel). n = 6 per group. (D) %FS in WT, non-treated TG, and CDS-treated TG mice at 24-weeks as evaluated with echocardiography. N = 6 per group. (E) Effects of CDS on increased mRNA expression of ANP, BNP and βMHC in the heart of TG mice at 24-weeks. N = 5 mice per group. (F) Phosphorylation of ERK in heart. The extracts of the hearts from WT, non-treated TG, and CDS-treated TG mice at 24-weeks was analyzed by Western blotting using anti-phospho ERK (p-ERK) antibody. n = 5 mice per group. (G) Effects of CDS on mRNA expression of IL-6 in the heart of TG mice at 24-weeks. n = 5 mice per group. In A-G, *, p<0.05, and **, p<0.01.

In TG mice hearts, the mRNA expression of angiotensin converting enzyme-1 (ACE) but not angiotensinogen, AT1 or AT2 was elevated compared with WT mice hearts (S4 Fig). Therefore, we determined the effect of pharmacological AT1 blockade on cardiac remodeling in TG mice. Administration of the AT1-selective antagonist candesartan (CDS), which started at 6-weeks, nearly abolished both cardiac hypertrophy and fibrosis with improvement of %FS at 24-weeks (Fig 3A–3D). The ACE inhibitor cilazapril also prevented cardiac hypertrophy in TG mice (S5 Fig). Consistent with the resolution of cardiac hypertrophy and fibrosis by blockade of the Ang II-AT1 pathway, the upregulation of mRNA expression of ANP, BNP, and β-MHC was all reversed by CDS (Fig 3E). Compared with WT mice hearts, TG mice hearts showed increased ERK phosphorylation, which was abolished by administration of CDS (Fig 3F).

### Cardiac hypertrophy induced by S1PR1 overexpression in fibroblasts is mediated through Ang II-dependent production of IL-6

The mRNA expression of IL-6, a hypertrophic mediator, in TG mice hearts was 5-fold increased with WT mice hearts (Fig 3G). In addition, the mRNA expression of leukemia inhibitory factor (LIF), another IL-6 family of cytokine, was slightly increased in TG mice hearts compared with WT mice hearts (Panel A in S6 Fig). In contract, cardiac mRNAs of cardiotrophin-1, the central signal-transducing receptor GP130 for IL-6 family cytokines, LIF receptor, insulin-like growth factor-I or TGF-β1 were not different between TG and WT mice (Panels A and B in S6 Fig). Notably, administration of CDS abolished upregulation of IL-6 mRNA in TG mice hearts (Fig 3G).

**Fig 4:**
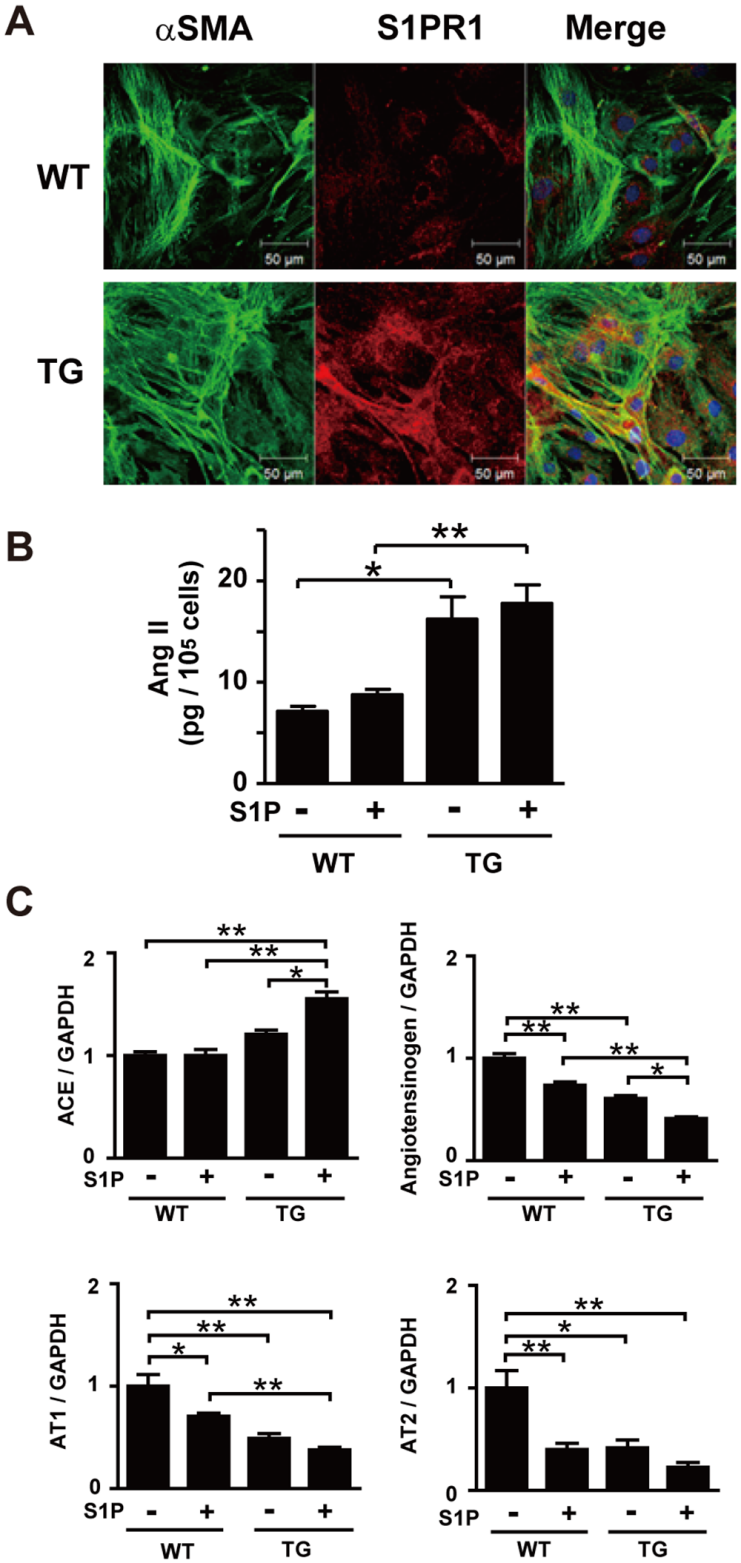
Cardiac fibroblasts overexpressing S1PR1 release a greater amount of Ang II. (A) Double immunofluorescence staining of fibroblasts isolated from 24-week-old WT and TG mice, using anti-αSMA and anti-S1PR1 antibodies. S1PR1 is more abundantly expressed in fibroblasts from TG mice compared with WT mice. (B) Ang II peptide levels in the conditioned media of WT and TG fibroblasts cultured in the presence and absence of S1P (100 nmol/L) without serum for 24 h. n = 6 per group. (C) Real-time PCR analysis of mRNAs of ACE, angiotensinogen, AT_1_ and AT_2_ in WT and TG fibroblasts. In B and C, *, p<0.05 and **, p<0.01.

We isolated fibroblasts from WT and TG mouse hearts. Nearly all cells from both WT and TG mice were positive for S1PR1 as well as αSMA (Fig 4A) with higher expression of S1PR1 in fibroblasts from TG mice. In contrast, the mRNA of S1PR1 transgene was hardly detectable in cardiomyocytes from TG mice (Panel B in S7 Fig). The mRNA expression of S1PR2 and S1PR3 was similar in fibroblasts from WT and TG mice hearts (Panel A in S7 Fig).

The level of Ang II peptide in the conditioned media of cardiac fibroblasts from TG mice was 2.5-fold greater compared with that in fibroblasts from WT mice (Fig 4B). S1P treatment of fibroblasts did not affect Ang II release. ACE mRNA levels were increased in S1P-treated, TG mouse-derived fibroblasts compared with WT fibroblasts whereas those of angiotensinogen, AT1 and AT2 were rather decreased in TG fibroblasts than in WT fibroblasts (Fig 4C).

**Fig 5:**
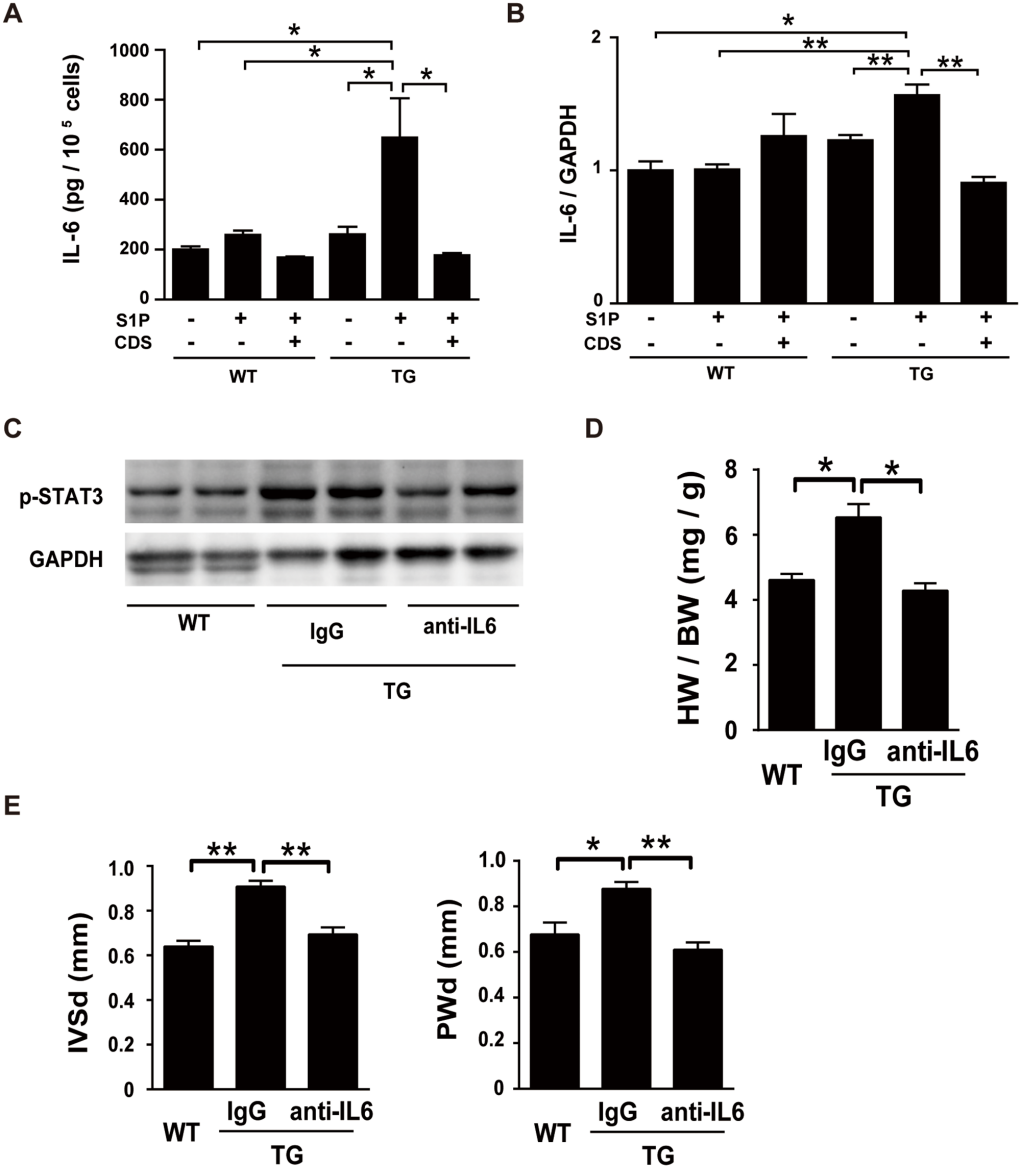
IL-6 is involved in cardiac hypertrophy in TG mice. (A) IL-6 peptide levels in the conditioned media of fibroblasts, which were isolated from 24-week-old WT and TG mice and cultured in the presence and absence of S1P (100 nmol/L) and CDS (1 μmol/L) without serum for 24 h. n = 8. (B) Expression of IL-6 mRNA in cardiac fibroblasts isolated from 24-week-old WT and TG mice. Fibroblasts were cultured in the presence and absence of S1P (100 nmol/L) and CDS (1 μmol/L) without serum for 24 h. n = 8. (C-E) Effects of administration (every two weeks) of neutralizing anti-IL-6 antibody or control isotype-matched IgG, which was started at 8-weeks, and mice were sacrificed at 12-weeks. (C) The effects of anti-IL-6 antibody administration on the phosphorylation of STAT3 were determined at 12-weeks. Mice received i.p. injections of anti-IL6 antibody or isotype—matched control IgG as described in Materials and methods. (D) Effects of anti-IL-6 antibody administration on the HW/BW ratio was determined at 12-weeks. n = 5 mice per group. (E) Effects of anti-IL-6 antibody administration on IVSd and PWd. Mice received anti-IL-6 antibody or control IgG as described in C, and underwent echocardiographic analysis at 12-week-old. n = 5 mice per group.

We next studied the effects of S1P and AT1 on IL-6 release from isolated cardiac fibroblasts. S1P treatment stimulated IL-6 release in TG fibroblasts but not WT fibroblasts (Fig 5A). The addition of CDS abolished S1P-induced stimulation of IL-6 release. S1P also increased IL-6 mRNA in TG fibroblasts but not WT fibroblasts, which was inhibited by the addition of CDS (Fig 5B). Finally, we tested the effects of administration of a specific IL-6 neutralizing antibody on cardiac hypertrophy and STAT3 phosphorylation in TG mice. The control TG mouse group received injections of isotype-matched control IgG. The tyrosine phosphorylation level of STAT3, a key signaling molecule downstream of IL6, in the heart was elevated in control IgG-given TG mice at 12-weeks compared with WT mice (Fig 5C). Administration of anti-IL-6 antibody abolished an increase in STAT3 phosphorylation in TG mouse heart. Anti-IL-6 antibody also abolished an increase in the heart weight (HW) / body weight (BW) ratio and the interventricular septum dimension (IVSd) and left ventricular posterior wall dimension (PWd) in TG mice at 12-weeks (Fig 5D and 5E).

## Discussion

This study demonstrates a novel fibroblast-mediated effect of S1PR1 on cardiac remodeling. Augmented S1PR1 action due to αSMA promoter-driven S1PR1 overexpression in mice leads to cardiac hypertrophy and fibrosis with reduced contractility in the absence of hemodynamic stress. In TG mouse heart, S1PR1 is overexpressed in hyperplastic fibroblasts/myofibrablasts but not cardiomyocytes. Mechanistically, S1PR1-mediated cardiac hypertrophy is characterized by total dependence on Ang II-AT1 axis and IL-6. Ang II-AT1 is involved in production of IL-6 in fibroblasts in a paracrine/autocrine manner.

During cardiac remodeling, fibroblasts in heart undergo the characteristic phenotypic changes to differentiate into myofibroblasts, which express smooth muscle-specific contractile protein αSMA [14]. We employed the αSMA promoter to overexpress S1PR1, a receptor which stimulates cell proliferation and migration in fibroblasts [4, 11]. As expected, the majority of CD31-negative and S1PR1-positive cells in TG mice hearts were also positive for the fibroblast/myofibroblast markers, αSMA and collagen I α-2 [9, 15]. The expression at a single cell level of S1PR1 as well as αSMA was more intense in fibroblasts from TG mice compared with WT mice, indicating that the αSMA promoter was active in fibroblasts from TG mice. Cardiac fibroblasts express a much higher level of sphingosine kinase-1 compared with cardiomyocytes [16], suggesting that S1PR1 in cardiac fibroblasts may be effectively activated by autocrine/paracrine S1P in TG mice. Consistent with these, fibroblast/myofibroblasts were hyperplastic in TG heart as indicated by the greater anti-αSMA positive cells. Thus, αSMA promoter-driven S1PR1 overexpression facilitated proliferation of cardiac fibroblasts with the myofibroblastic phenotype in TG mice.

Cardiac fibroblasts regulate phenotypes of cardiomyocytes and fibroblasts themselves through releasing a variety of paracrine humoral factors including Ang II, TGF-β, IGF-I and IL-6 [17]. A recent study [18] revealed that fibroblasts play an essential role in hypertrophic response to pressure overload by releasing IGF-I. Cardiac fibroblasts have all the components of the renin-angiotensin system (RAS) [19]. In the present study, fibroblasts from TG mouse heart expressed a higher mRNA level of ACE and produced a larger amount of Ang II, which was crucially involved in cardiac remodeling in TG mice via AT1. Because Ang II induces upregulation of S1PR1 (S1 Fig), there may be an autocrine/paracrine positive feedback loop comprising S1PR1-Ang II-AT1. Besides Ang II, we observed 3 to 5 fold increase in IL-6 mRNA in TG mouse heart and S1P-induced IL-6 peptide release from TG fibroblasts compared with WT mice. None of the other hypertrophic mediators and their receptors examined was not elevated, except LIF which showed a slight increase in TG heart. Notably, the increase in IL-6 mRNA expression in TG mouse heart was dependent on AT1. IL-6 has a hypertrophic activity for cardiomyocytes both in vitro and in vivo [20, 21], and is involved in exogenous Ang II-induced cardiac hypertrophy [22]. These previous observations suggest that IL-6 is a downstream effector of Ang II signaling to mediate cardiac hypertrophy. In the present study, blockade of IL-6 action by the neutralizing antibody abolished hypertrophy in TG mice. Therefore, it is likely that augmented S1PR1 action in fibroblasts stimulated release of Ang II and thereby IL-6, culminating in hypertrophy. Alternatively, it might also be possible that S1PR1 and AT1 could form the positive feedback loop to stimulate each other or that S1PR1 and AT1 could operate in parallel pathways, which are both necessary for the development of the hypertrophic phenotype in TG mice, with the interaction of stimulating each pathway. Further study is required for fully understanding the interplays of S1PR1 and AT1.

We observed that TG mouse-derived fibroblasts showed enhanced Ang II production under the serum-free culture condition, compared with wild-type fibroblasts (Fig 4B). Differently from the case of IL-6 production, which will be discussed below, in vitro treatment of the cells with exogenous S1P did not increase Ang II production in TG fibroblasts. Fibroblasts from TG mice hearts exhibited enhanced differentiation toward myofibroblasts as demonstrated by increased αSMA expression (Fig 2). The upregulated αSMA expression in fibroblasts was maintained at least for the three passages of culture in fibroblasts isolated from TG mouse heart. Cultured fibroblasts from TG mouse heart also showed upregulation of ACE gene expression (Fig 4C). Thus, cardiac fibroblasts isolated from TG mice maintains the phenotypic changes including altered gene expression of αSMA and ACE in vitro after isolation, suggesting that cardiac fibroblasts have undergone the S1PR1-mediated phenotypic changes in vivo. The in vivo phenotypic changes of cardiac fibroblasts may involve the actions of paracrine and autocrine humoral factors including angiotensin II, IL-6 cytokine family members, TGFβ and IGF-I (Figs 3–5, S5 and S7 Figs). Alternatively, it is possible that even a low concentration of S1P, which was released into the media from fibroblasts, could be enough to maintain the phenotype of increased angiotensin II production.

IL-6 production is driven mainly by two intracellular signaling pathways, STAT3 and NF-κB pathways. S1PR1 in tumor cells was previously shown to be linked to STAT3 activation, which contributed to stimulated IL-6 production [23] [24]. Therefore, it is an interesting possibility that S1P stimulation of IL-6 production in TG fibroblasts but not WT fibroblasts (Fig 5A) could be mediated through S1PR1-STAT3 pathway. Importantly, stimulation of IL-6 release by S1P is dependent on AT1. AT1 was previously shown to couple to phosphorylation of STAT1 and STAT2 via tyrosine kinase JAK2 [25]. Phosphorylated STATs homo-dimerize or hetero-dimerize and exhibit isoform-specific activities. It may be possible that differential activation of STAT family members by S1PR1 and AT1 could underlie the cooperative actions of S1PR1 and AT1 in stimulating IL-6 production. The precise molecular mechanisms of S1PR1- and AT1-dependent IL-6 release, and possible functional cross talk between S1PR1 and AT1R remain to be clarified.

These are several genetically modified animal models for cardiac hypertrophy, in which the cardiac RAS is targeted [26]. They are largely the mouse models that aimed at overproducing Ang II or overexpressing AT1 in cardiomyocytes. The αMHC promoter-driven overexpression of angiotensinogen, which resulted in a 2-fold increase in cardiac Ang II level, was accompanied by bi-ventricular hypertrophy and age-dependent cardiac dysfunction but not fibrosis [27]. In contrast, αMHC promoter-driven overexpression of ACE [28] or an engineered fusion protein designed to directly release Ang II [29] in cardiomyocytes did not result in ventricular hypertrophy despite of greater increases in cardiac Ang II level compared with the above mentioned study [27]. The effects of αMHC promoter-driven overexpression of AT1 are also complicated; transgenic overexpression of AT1 in cardiomyocytes, only when overexpressed at very high levels, e.g. 200-fold compared with WT mice, resulted in ventricular hypertrophy [30]. The observations may suggest that increased Ang II generation and AT1 expression in cardiomyocytes could be rather the limiting factor than the primary determinant in RAS-mediated hypertrophic effects. Our model is distinct from these genetically modified animal models in that augmented activation of S1PR1 in fibroblasts results in stimulation of Ang II generation in fibroblasts. Our model is the first transgenic mouse model in which stimulation of local RAS in fibroblasts results in ventricular hypertrophy and fibrosis through S1PR1- and AT1-dependent IL-6 release from fibroblasts. This Ang II-dependent remodeling model spares other organs and tissues including lung, liver and arteries although the mechanism for this sparing is unknown presently.

In our recent study [8], we demonstrated that transgenic overexpression of SphK1 in mice resulted in interstitial cardiac fibrosis but not hypertrophy, which was mediated via S1PR3 but independent of AT1. S1PR3 signaling in cardiac fibrosis involved reactive oxygen species generation and TGF-β-Smad2/3 pathway. Cardiac fibroblasts endogenously express S1PR3 and S1PR2, besides S1PR1 [8, 11]. Therefore, it is possible that *in vivo* stimulation of fibroblast S1P receptors in SphK1-transgenic mouse heart might result in relatively dominant activation of S1PR3, which is different from the present case of transgenic S1PR1-overexpression. Activation of S1PR3, which couples to Gq and G12/13 unlike S1PR1, might lead to cardiac fibrosis without hypertrophy. Alternatively, increased cardiac S1P level in SphK1-transgenic mice activates multiple S1P receptors in both fibroblasts and myocytes, which might bring about the different outcome from the case of augmented S1PR1 activation in fibroblasts in S1PR1-transgenic mice. Besides causing cardiac fibrosis, SphK1 overexpression also exerted a protective effect from ischemia/reperfusion injury, which was shown to be mediated by S1PR2 and S1PR3 [8, 11, 31]. The effect of S1PR1 overexpression in fibroblasts against cardiac injury deserves further investigation.

This study may have the following limitations; 1) the αSMA promoter drives gene expression in smooth muscle of various organs in adult mice, besides myofibroblasts. Therefore, the possibility that other αSMA-positive cells to express S1PR1 transgene than myofibroblasts could be involved in the development of the phenotype in S1PR1-TG mice cannot be completely excluded. 2) this study is based on the analyses in S1PR1-transgenic mouse model. Another loss of function mutation approach using fibroblast-specific S1PR1 gene deletion would be informative in understanding a role of S1PR1 in cardiac hypertrophy and may provide a new insight into therapeutic clues for preventing cardiac fibrosis.

Our data show the crucial role of S1PR1-mediated lysophospholipid signaling in cardiac fibroblasts for cardiac hypertrophy and fibrosis. This S1PR1 action involves stimulation of local RAS activity and thereby IL-6 generation on fibroblasts. These findings reveal the novel molecular link for engagement of fibroblasts in cardiac remodeling, pointing to a new therapeutic approach for cardiac remodeling. Our TG mice also provide a useful animal model for studying pathological cardiac hypertrophy, particularly the role of local RAS in cardiac remodeling.

## Supporting information

S1 Fig Ang II infusion induces hypertrophy and an increase in mRNAs of S1PR1 and sphingosine kinase-1 in the heart of mice infused with Ang II. Ang II was infused with osmotic mini-pump for 14 days. (A) The heart weight / body weight (HW / BW) ratio in mice receiving vehicle (saline) and Ang II. n = 5 mice per group. (B) Real-time PCR analysis of mRNAs of S1P receptors, SphKs and aSMA in the hearts of mice. n = 5 mice per group. In A and B, * p<0.05, ** p<0.01.
(TIF)

S2 Fig Expression of S1PR1 transgene and endogenous S1P receptors in WT and TG mice. (A) Northern blot analysis of S1P receptors in various organs. S1PR1 transgene is readily detected in lung, intestine, heart, kidney and brain. There is no difference in the endogenou gene expression of S1PR1, S1PR2 and S1PR3 between WT and TG mice. (B) Expression of S1PR1 transgene and endogenous gene in hearts of three TG mouse lines. Lines 4 and 11 abundantly express S1PR1 transgene whereas line 5 modestly expressed S1PR1 transgene. (C) Protein expression of S1PR1 and GAPDH (glyceraldehyde 3-phosphate dehydrogenase) in heart was determined by western blotting using anti-S1PR1 and anti-GAPDH antibodies. (D-G) S1PR1 was overexpressed in vascular smooth muscle cells (arrows) and interstitial cells (arrowhead) in the heart of TG mice. (H) Aortic media, bronchus, intestine, urinary bladder and uterus. S1PR1 was overexpressed in the smooth muscle layers of these organs in TG mice.
(TIF)

S3 Fig Echocardiographic analysis of hypertrophic hearts from WT and TG mice. (A) end-diastolic interventricular septal dimension (IVsd). (B) end-diastolic posterior wall dimension (PWd). (C) end-diastolic left ventricular diameter (EDD). (D) %fractional shortening (% FS). n = 7~8 mice per group. * P<0.01.
(TIF)

S4 Fig Cardiac mRNA expression of angiotensin signaling system in WT and TG mice. Real-time PCR analysis of mRNAs of angiotensinogen, ACE, AT1 and AT2 in WT and TG hearts. n = 5 mice per group. * p<0.05.
(TIF)

S5 Fig Blockade of angiotensin system prevents cardiac hypertrophy and fetal gene expression in TG mice. The ACE inhibitor cilazapril were administered into mice as described in Methods, and mice were analyzed at 24 weeks. Effect of cilazapril on the HW / BW ratio in TG mice. n = 5 mice per group. * p<0.05.
(TIF)

S6 Fig Expression of hypertrophic mediators and receptors in the heart of WT and TG mice and effects of an AT1 antagonist on their expression. Expression of mRNAs were analyzed by real-time PCR. (A) Expression of mRNAs of cardiotrophin1, LIF, GP130 and LIFR in the hearts of WT and TG mice. (B) Effects of CDS on mRNA expression of endothelin1, IGF-I and TGFβ in the heart of TG mice. n = 5 mice per group. n = 5 mice per group. In (A) and (B), * p<0.05.
(TIF)

S7 Fig Expression of S1PR1 in cardiac fibroblasts and cardiomyocytes. (A) Expression of S1PR1, S1PR2 and S1PR3 in cardiac fibroblasts isolated from WT and TG mice. The expression of S1P receptor mRNAs Total RNA was determined by reverse transcription-PCR. (B) The expression of endogenous S1PR1, S1PR1 transgene and internal control GAPDH was determined by Northern blotting. Total RNA was isolated from cardiomyocytes and heart tissues.
(TIF)

S1 Table Characteristics of WT and TG mice. (TIF)
