# Supplementary figures and images for: Augmented sphingosine 1 phosphate receptor-1 signaling in cardiac fibroblasts induces cardiac hypertrophy and fibrosis through angiotensin II and interleukin-6
Source: PLoS One. 2017 Aug 3;12(8):e0182329. doi: 10.1371/journal.pone.0182329 (PMC5542600; doi:10.1371/journal.pone.0182329)

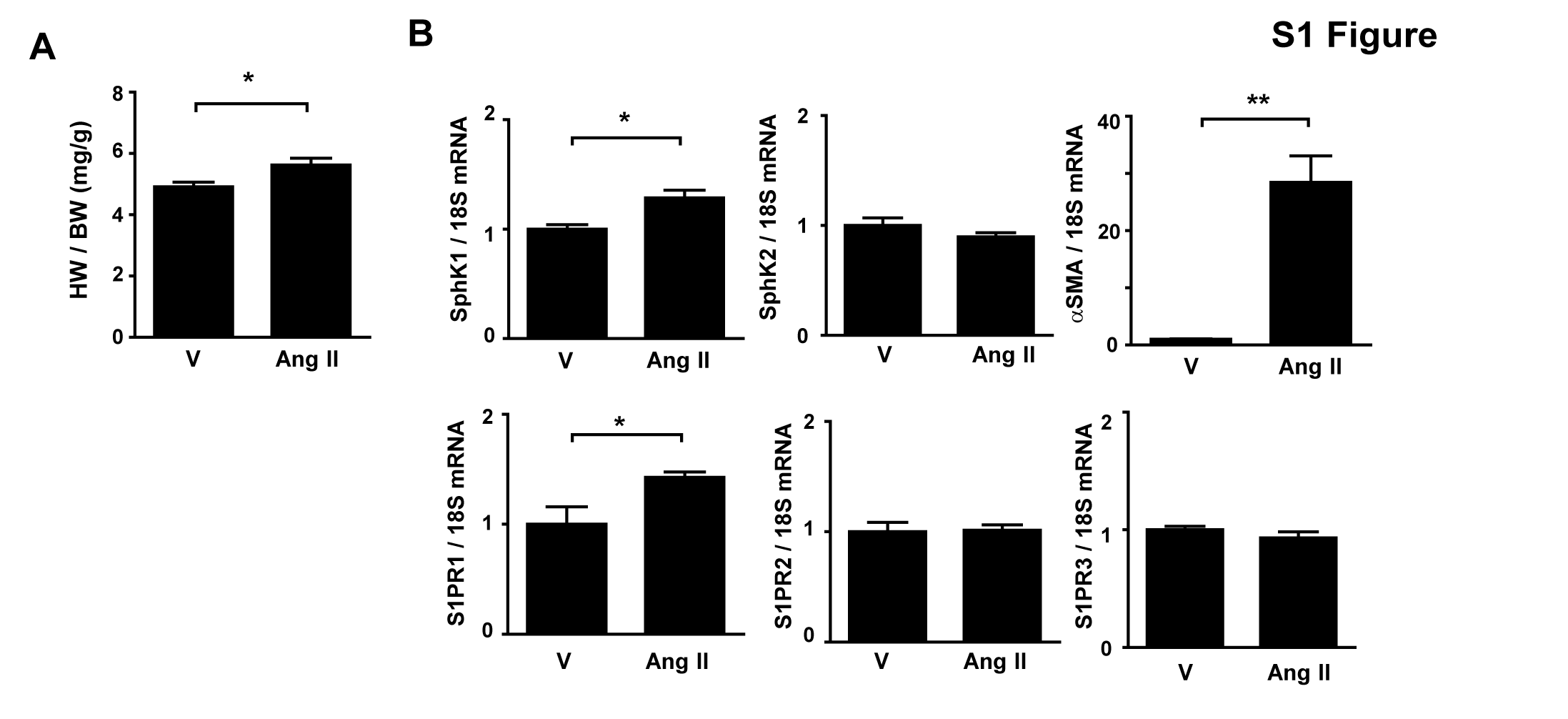

Supplement: S1 Fig — Ang II was infused with osmotic mini-pump for 14 days. (A) The heart weight / body weight (HW / BW) ratio in mice receiving vehicle (saline) and Ang II. n = 5 mice per group. (B) Real-time PCR analysis of mRNAs of S1P receptors, SphKs and aSMA in the hearts of mice. n = 5 mice per group. In A and B, * p<0.05, ** p<0.01. (TIF) [file pone.0182329.s001.tif]

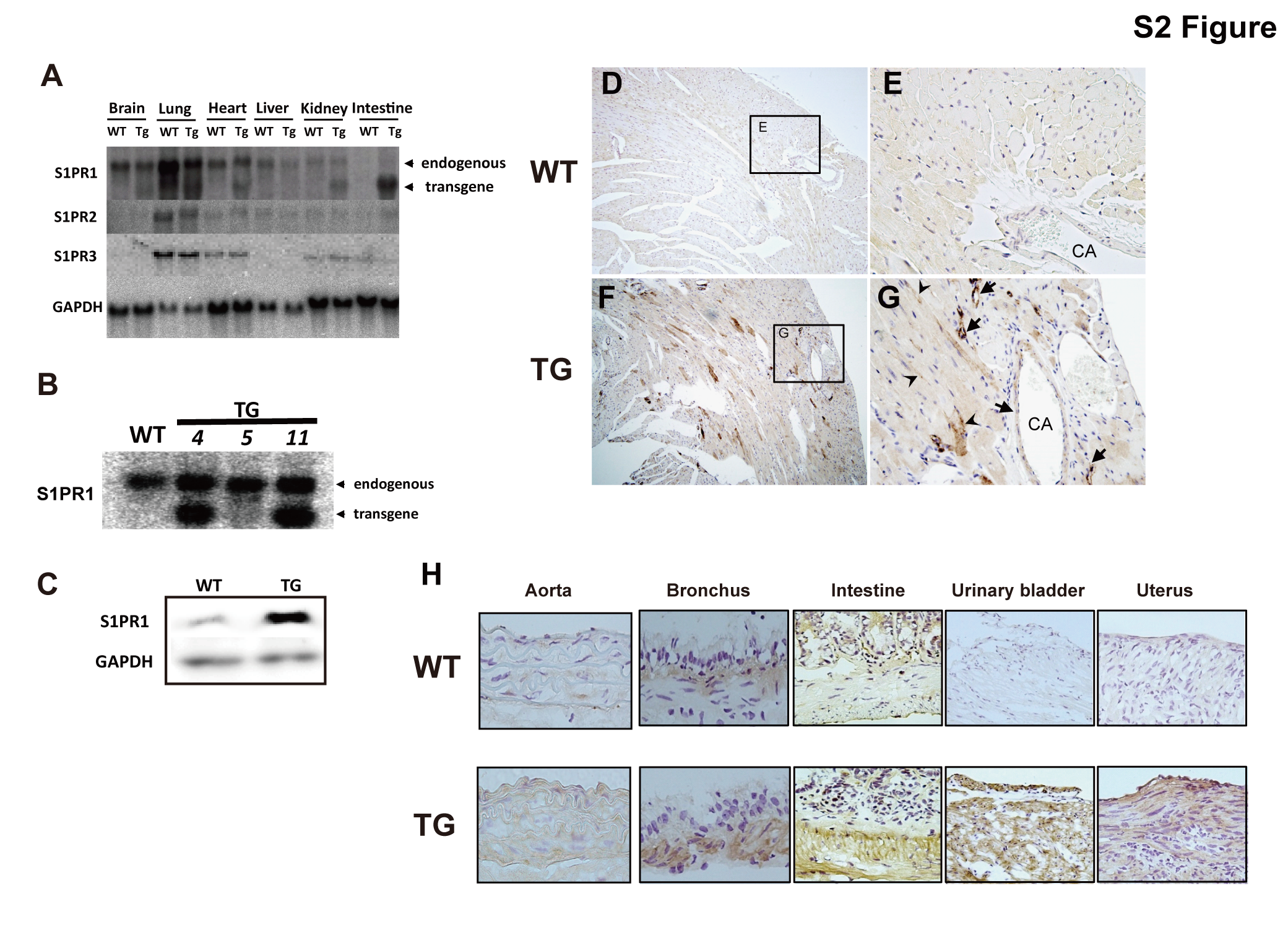

Supplement: S2 Fig — (A) Northern blot analysis of S1P receptors in various organs. S1PR1 transgene is readily detected in lung, intestine, heart, kidney and brain. There is no difference in the endogenou gene expression of S1PR1, S1PR2 and S1PR3 between WT and TG mice. (B) Expression of S1PR1 transgene and endogenous gene in hearts of three TG mouse lines. Lines 4 and 11 abundantly express S1PR1 transgene whereas line 5 modestly expressed S1PR1 transgene. (C) Protein expression of S1PR1 and GAPDH (glyceraldehyde 3-phosphate dehydrogenase) in heart was determined by western blotting using anti-S1PR1 and anti-GAPDH antibodies. (D-G) S1PR1 was overexpressed in vascular smooth muscle cells (arrows) and interstitial cells (arrowhead) in the heart of TG mice. (H) Aortic media, bronchus, intestine, urinary bladder and uterus. S1PR1 was overexpressed in the smooth muscle layers of these organs in TG mice. (TIF) [file pone.0182329.s002.tif]

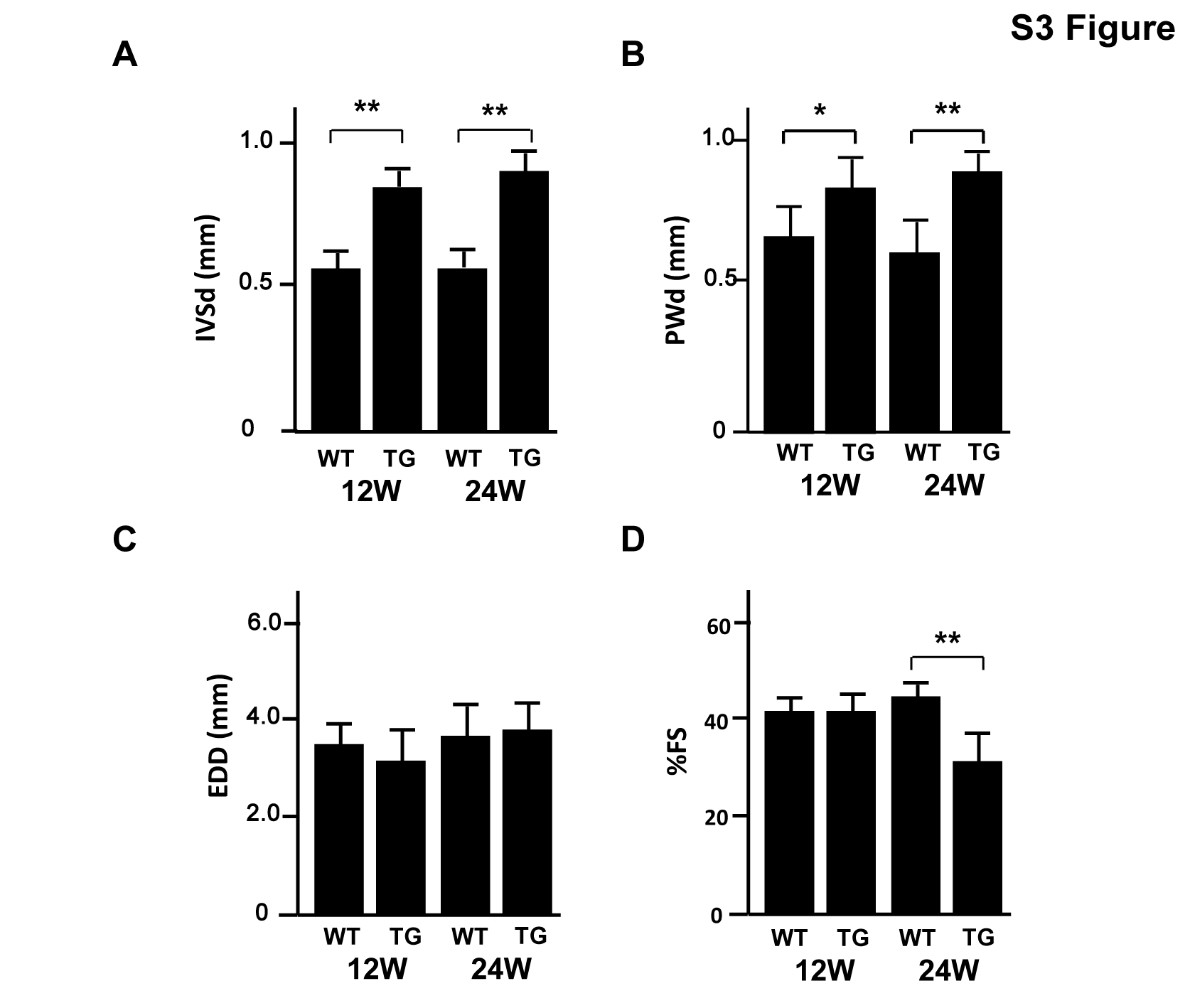

Supplement: S3 Fig — (A) end-diastolic interventricular septal dimension (IVsd). (B) end-diastolic posterior wall dimension (PWd). (C) end-diastolic left ventricular diameter (EDD). (D) %fractional shortening (% FS). n = 7~8 mice per group. * P<0.01. (TIF) [file pone.0182329.s003.tif]

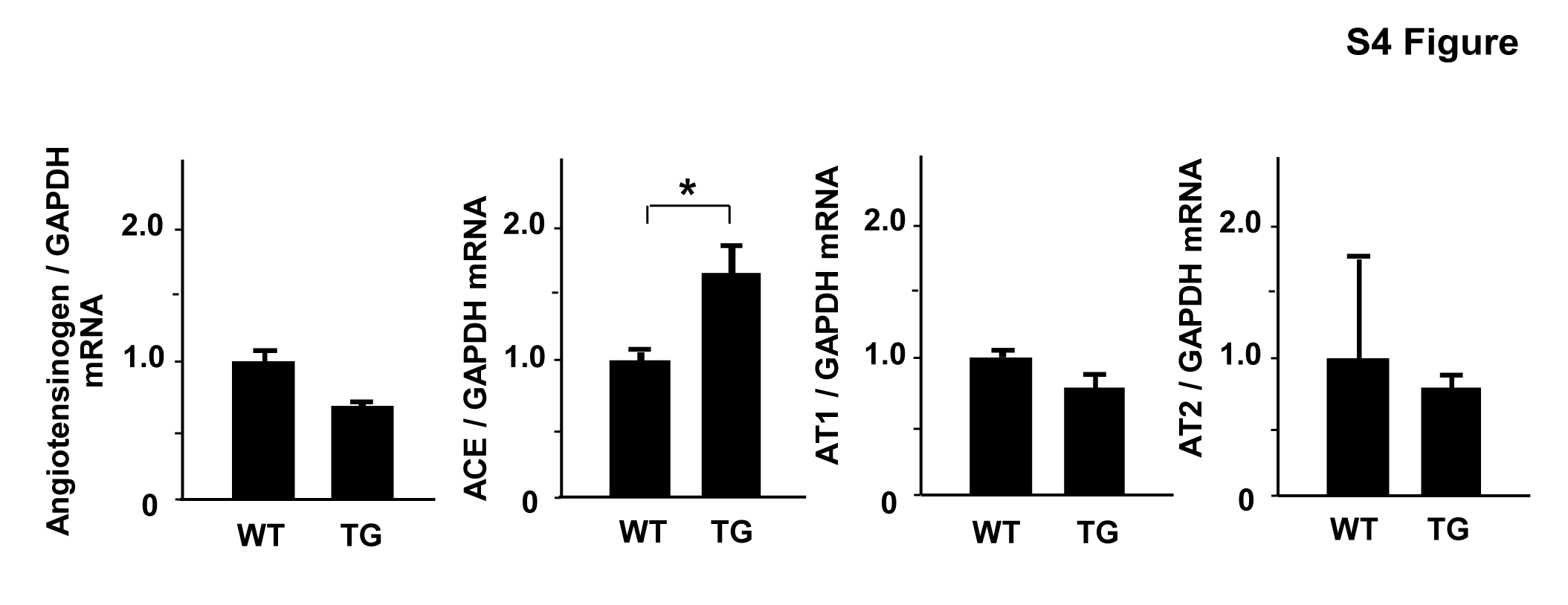

Supplement: S4 Fig — Real-time PCR analysis of mRNAs of angiotensinogen, ACE, AT1 and AT2 in WT and TG hearts. n = 5 mice per group. * p<0.05. (TIF) [file pone.0182329.s004.tif]

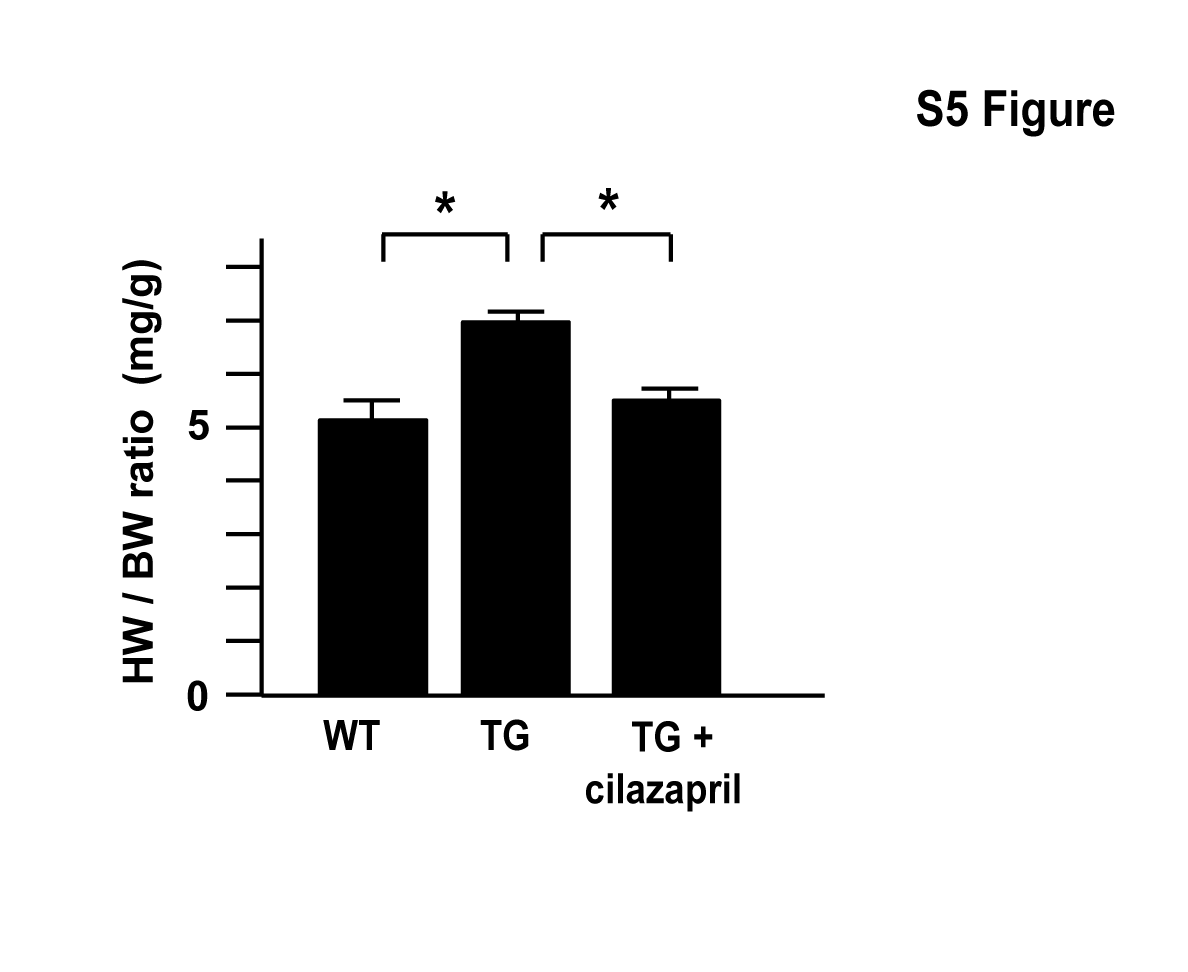

Supplement: S5 Fig — The ACE inhibitor cilazapril were administered into mice as described in Methods, and mice were analyzed at 24 weeks. Effect of cilazapril on the HW / BW ratio in TG mice. n = 5 mice per group. * p<0.05. (TIF) [file pone.0182329.s005.tif]

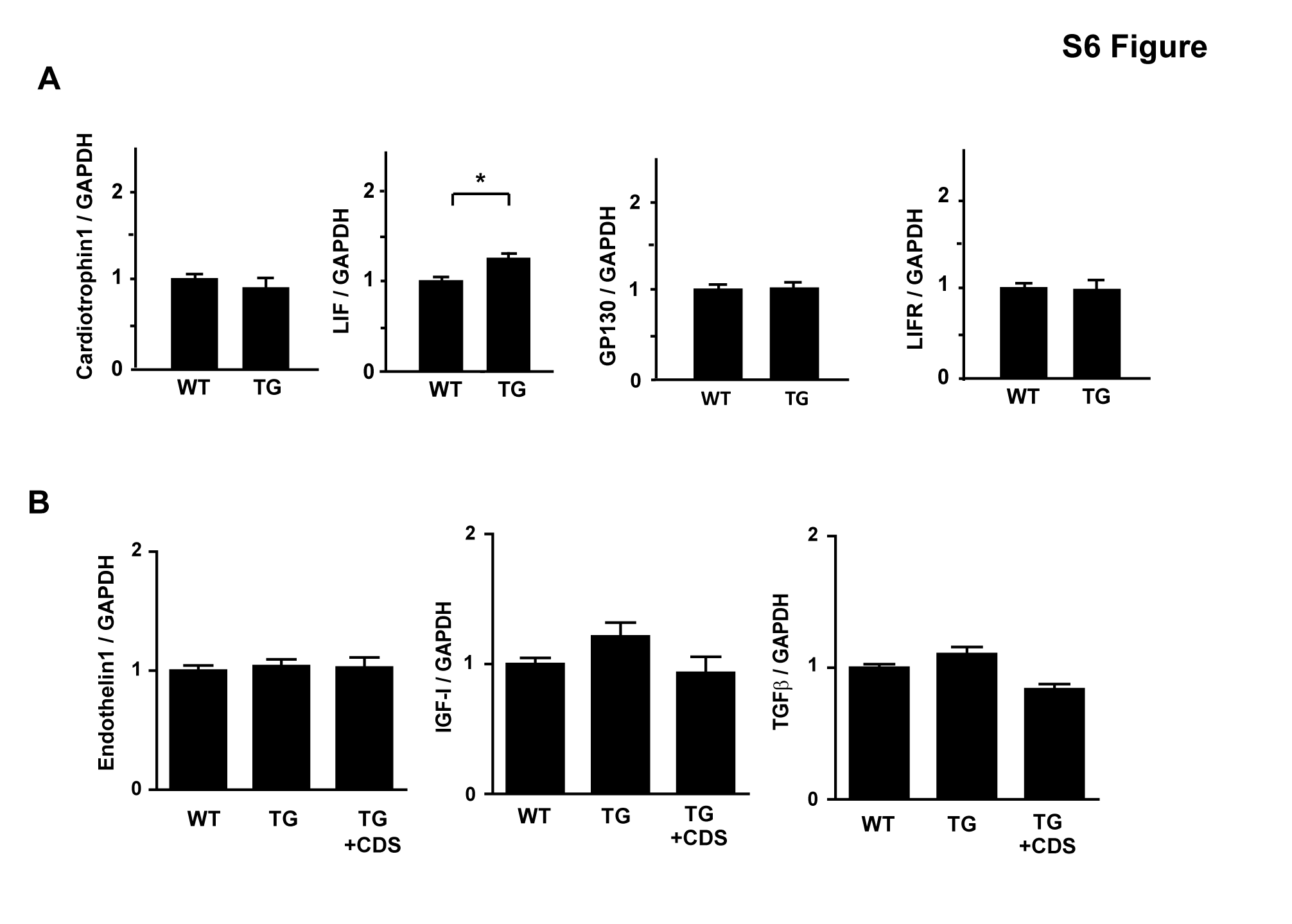

Supplement: S6 Fig — Expression of mRNAs were analyzed by real-time PCR. (A) Expression of mRNAs of cardiotrophin1, LIF, GP130 and LIFR in the hearts of WT and TG mice. (B) Effects of CDS on mRNA expression of endothelin1, IGF-I and TGFβ in the heart of TG mice. n = 5 mice per group. n = 5 mice per group. In (A) and (B), * p<0.05. (TIF) [file pone.0182329.s006.tif]

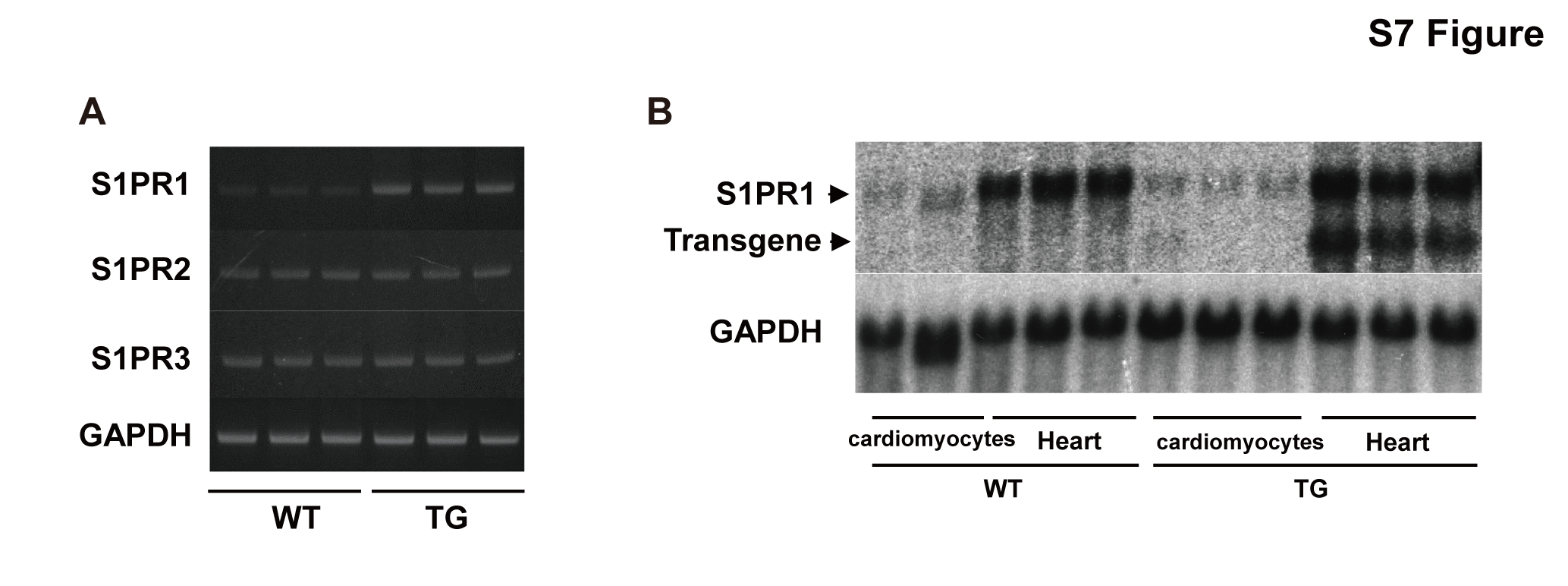

Supplement: S7 Fig — (A) Expression of S1PR1, S1PR2 and S1PR3 in cardiac fibroblasts isolated from WT and TG mice. The expression of S1P receptor mRNAs Total RNA was determined by reverse transcription-PCR. (B) The expression of endogenous S1PR1, S1PR1 transgene and internal control GAPDH was determined by Northern blotting. Total RNA was isolated from cardiomyocytes and heart tissues. (TIF) [file pone.0182329.s007.tif]

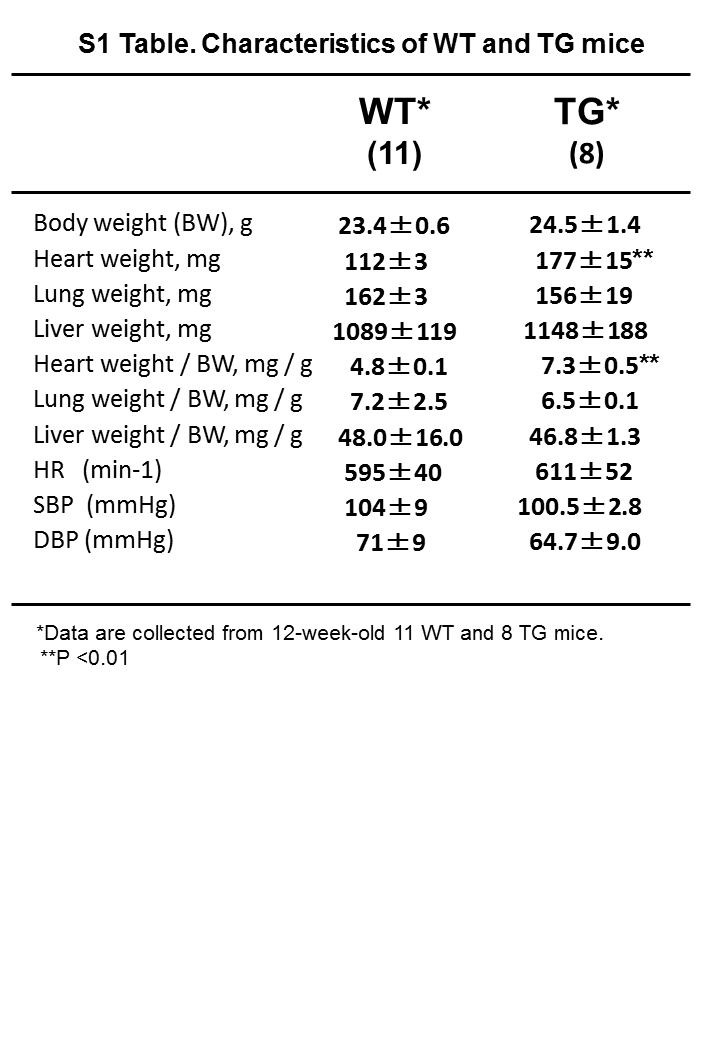

Supplement: S1 Table — (TIF) [file pone.0182329.s008.tif]
